# Flexible computation offloading in a fuzzy-based mobile edge orchestrator for IoT applications

**DOI:** 10.1186/s13677-020-00211-9

**Published:** 2020-11-25

**Authors:** VanDung Nguyen, Tran Trong Khanh, Tri D. T. Nguyen, Choong Seon Hong, Eui-Nam Huh

**Affiliations:** Department of Computer Science and Engineering, Kyung Hee University, Korea, Deokyoungdaero, Yongin, Korea

**Keywords:** Internet of Things, Fuzzy logic, Mobile edge orchestrator, Offload decision

## Abstract

In the Internet of Things (IoT) era, the capacity-limited Internet and uncontrollable service delays for various new applications, such as video streaming analysis and augmented reality, are challenges. Cloud computing systems, also known as a solution that offloads energy-consuming computation of IoT applications to a cloud server, cannot meet the delay-sensitive and context-aware service requirements. To address this issue, an edge computing system provides timely and context-aware services by bringing the computations and storage closer to the user. The dynamic flow of requests that can be efficiently processed is a significant challenge for edge and cloud computing systems. To improve the performance of IoT systems, the mobile edge orchestrator (MEO), which is an application placement controller, was designed by integrating end mobile devices with edge and cloud computing systems. In this paper, we propose a flexible computation offloading method in a fuzzy-based MEO for IoT applications in order to improve the efficiency in computational resource management. Considering the network, computation resources, and task requirements, a fuzzy-based MEO allows edge workload orchestration actions to decide whether to offload a mobile user to local edge, neighboring edge, or cloud servers. Additionally, increasing packet sizes will affect the failed-task ratio when the number of mobile devices increases. To reduce failed tasks because of transmission collisions and to improve service times for time-critical tasks, we define a new input crisp value, and a new output decision for a fuzzy-based MEO. Using the EdgeCloudSim simulator, we evaluate our proposal with four benchmark algorithms in augmented reality, healthcare, compute-intensive, and infotainment applications. Simulation results show that our proposal provides better results in terms of WLAN delay, service times, the number of failed tasks, and VM utilization.

## Introduction

Recently, fifth-generation (5G) cellular technologies have enabled various new applications. such as video streaming analysis, augmented reality (AR), the Internet of Things (IoT), and autonomous driving [1]. These applications have become significantly evolved and diverse. Key applications of the IoT are AR and virtual reality (VR), which are among the most promising applications for smartphone users and autonomous vehicles [2]. Moreover, the era of the IoT implies a large number of sensors, actuators, and mobile devices deployed at the network edge. A considerable number of the computing tasks generated by these devices require timely, context-aware processing, and large amounts of computational resources, which current mobile devices lack. As a result, processing massive amounts of data traffic, and the growing demands for high data rates and computational capabilities are key features of the future Internet [3].

In order to enhance computation capabilities and reduce computation latency, mobile cloud computing systems have been proposed to enable mobile devices to utilize the powerful computing capability of the cloud [4–6]. Mobile users can access cloud computing over a wide area network (WAN) to process elastic services and conduct data-intensive analysis. However, in the IoT era, cloud computing cannot satisfy the service demands from the new trend of delay-sensitive applications. The reasons are as follows. First, WAN latency is high [7]. Second, the traffic capacity of a WAN will be significantly challenged by the dramatically increasing amount of data generated by IoT devices [4]. Finally, cloud computing works in a remote and centralized computing way; therefore, it cannot support context-aware computing for IoT applications [8].

Mobile edge computing (MEC) addresses these challenges by bringing cloud servers closer to mobile devices at the edge of mobile networks. Moreover, MEC standardization was introduced by the European Telecommunications Standards Institute (ETSI) Industry Specification Group, and is a key enabler for 5G [9]. MEC offers an ultra–low latency environment with high bandwidth and real-time access to radio and network analytics. Therefore, MEC can provide latency-sensitive applications, and can reduce traffic bottlenecks in the core and backhaul networks while assisting in the offloading of heavy computational tasks from mobile devices to the edge [10]. The application task in MEC can move toward the edge and locally process data in proximity to the users [10]. First, mobile devices can access local computational resources using a wireless local area network (WLAN). Second, computation tasks of mobile devices can be processed or filtered before sending them to the cloud. However, the IoT environment opens up an innovation potential for efficient resource management that is mandatory to satisfy the quality requirements of different IoT application types, such as how the edge system responds to the dynamic flow of requests [11].

**Fig. 1:**
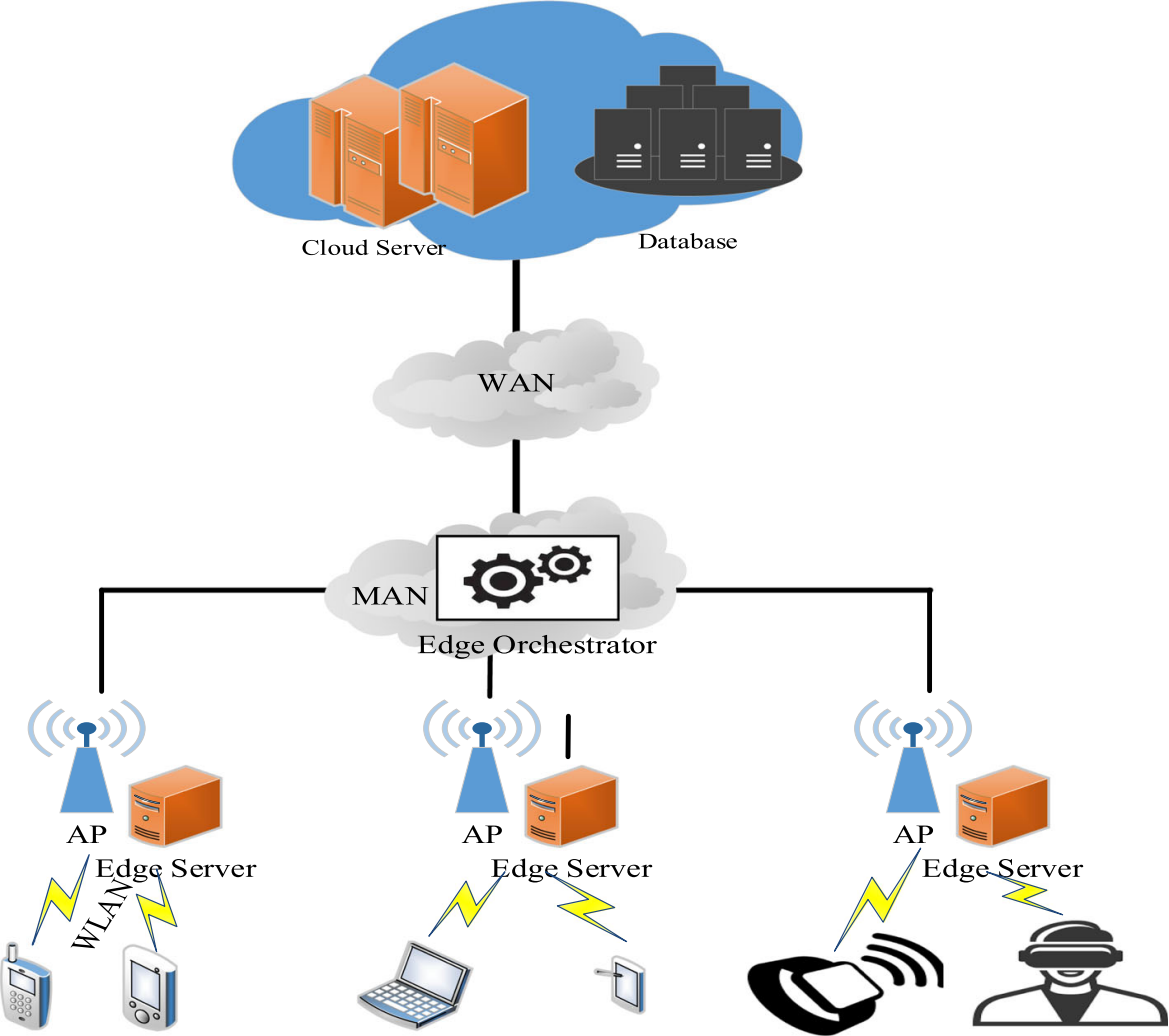
MEC and the role of edge orchestrator

One such approach is known as the mobile edge orchestrator (MEO) proposed by ETSI [9]. It is designed to integrate end devices, edge servers, and the cloud to form a hierarchical IoT architecture [3]. We depict this system in Fig. 1. The MEO can manage the available computing/storage/network resources and the life cycle of the application [12]. The MEO decides the destination computational unit for each offloaded task within edge computing.

Two major actions in the MEO are monitoring the rapidly changing network conditions and determining the target VMs for offloading. Many approaches have been proposed for solving the workload orchestration problem. The decisions made can be based on various conditions, such as a combination of network latency, data transfer size, and remote server capabilities [13]. Based on the WLAN, the metropolitan area network (MAN), and WAN communications, and the requirements of the application task, the decision can be made based on the edge/fog computing infrastructure [14]. However, these approaches did not study network congestion.

Fuzzy logic has recently become a good alternative to controlling real-world imprecision of rapidly changing uncertain systems, where it is difficult to provide accurate mathematical models. Because the fuzzy logic–based approach has lower computational complexity than other decision-making algorithms, it is significant for solving online and real-time problems. The decisions based on fuzzy logic are made considering edge and cloud computing characteristics [15]. Considering parameter link delay and the signal-to-noise ratio, the fuzzy-based offloading ratio was calculated in [16]. On the other hand, by using network information and matching it with the requirements received from applications, a fuzzy logic–based orchestrator can execute processes so the target mobile edge (ME) host can process applications [17]. In fuzzy-based MEO, the input variables chosen have a significant effect on system task execution. Additionally, the role of a fuzzy-based MEO is to find a target server that can be a local edge server, a neighboring edge server, or a cloud server based on the profile of an incoming application task and mobile edge computing characteristics. However, this system did not study the packet success ratio in the WLAN environment and the resource capability of a mobile device. Moreover, optimal mapping from service requirements to resource allocation, flexible inter-domain resource management for service delivery is open issue [18].

In our study, we investigate the MEO system and the WLAN environment. Therefore, we propose a new input variable and decision for a fuzzy inference system. Based on that, our proposal can find a target server for an incoming application task: either a mobile device, a local edge server, a neighboring edge server, or a cloud server. Moreover, the WLAN delay in our system also decreases. The key contributions of this paper are as follows.

1. We propose a new fuzzy inference system in order to decide where to offload the incoming task: its own resources, a local edge server, or a remote server.
2. We study the impact of packet length in the WLAN environment in terms of packet success ratio. Consequently, the resource capability of a mobile device is chosen based on the transmission time for the application task.
3. Based on the available information on the network connections and the states of the edge and the cloud, the MEO allows the mobile device to dynamically take the offloading decision: the edge or the cloud or the device itself. Therefore, it can improve the WLAN delay, service times, failed-task ratio, and VM utilization.
4. Our proposal provides better performance when the system is used for four applications under consideration: augmented reality (AR), healthcare, compute-intensive, and infotainment applications.

The remainder of this paper is organized as follows. “Related works” section reviews the related works. “Flexible computation offloading in a fuzzy-based mobile edge orchestration” section introduces the system model. “Performance evaluation” section describes flexible computation offloading in fuzzy-based mobile edge orchestration. “Conclusions” section provides the simulation results, followed by a conclusion and future research.

## Related works

The mobile edge orchestrator that is the central function in the MEC system maintains all information on MEC, such as all the deployed ME hosts, the services and resource availability in each host, the applications that are instantiated, and the topology of the network [19]. The MEO is basically used to manage and control the available computing, storage, network resources, and the requirements of incoming application tasks, and it maintains a catalogue of the applications that are available [9]. The MEO uses the requirements received from the application task for the decision on where to offload the process, and the target ME host. The basic decision method is that by matching the resource information with the application requirements, the MEO can select the target ME host [20]. To implement of MEO, Kristiani et al. [21] built a set of an intelligent air-quality monitoring system in Tunghai University. The authors use the Ganglia monitoring system to collect relevant information such as CPU, memory, network to monitor the power consumption and make a measurement and evaluation for Kubernetes Pods [21].

Considering MEO deployment, there are many approaches to solving where to deploy an incoming application task, as shown in Table 1. Baktir et al. [22] proposed a server-centric method by using orchestration of software-defined networking (SDN) on the edge server. In an edge computing system, Hegyi et al. [20] optimized placing the components of IoT applications, and Karagiannis et al. [23] proposed placement based on the partitioned shortest path (PSP) method to solve the placement optimization problem. In fog computing infrastructures, Santoro et al. [24] proposed efficient and optimized use of the infrastructure while satisfying the application requirements, naming the approach Foggy. On the other hand, Bittencourt et al. [14] proposed the edge-ward placement algorithm, where modules of the same application are grouped to be placed in the same device. This algorithm was designed according to three different policies: delay-sensitive versus delay-tolerant, resource management, and allocation. The results in this paper conclude that scheduling strategies can be designed to cope with different incoming application classes to take advantage of both fog and cloud computing characteristics [14].

**Table 1:** Comparison of MEO systems used in difference environments

	Method	MEO deployment	Key performance indicator
Baktir et al. [22]	IP-addressed and load-balancing	Edge server	Capabilities of SDN
Bittencourt et al. [14]	Edge-ward placement algorithm	Edge/fog computing infrastructures	CPU capacity and a static network delay for
			WLAN, MAN, and WAN communication
Hegyi et al. [20]	Optimize placing the components of	Edge server	Available virtualized resources
	IoT applications		
Karagiannis et al. [23]	PSP method solving placement optimization problem	Edge server	The provisioning of resources,
			replication degree of the applications
Santoro et al. [24]	Foggy: efficient and optimized use of the infrastructure	Fog computing infrastructures	Traditional and non-traditional requirements
	while satisfying the application requirements		

In the MEO, the offloading decision is made by taking many parameters into consideration, as shown in Table 1. As an example, the decision was made in [14] by the edge-ward placement algorithm based on CPU capacity and static network delay for WLAN, MAN, and WAN communication. The PSP [23] approach is based on the provisioning of resources and replication degree of the applications.

On the other hand, the fuzzy logic approach used in decision problems demonstrated that it can provide better results than classic algorithms [15, 17, 25, 26]. In [15], a fuzzy decision engine for code offloading considered both mobile and cloud variables. The script input parameters are CPU capacity, data length, video quality, and speed in the fuzzy logic system. The script output is a local or a remote processing decision. To determine a ratio for the user, the script input parameters used are parameter link delay and signal-to-noise ratio [25]. In [26], Rathore et al. solved multi-criteria decision-making problems to select an appropriate security service per mobile user requirements in fog and mobile edge computing by using a soft hesitant fuzzy rough set.

In the MEO, fuzzy logic is suitable for addressing technical challenges against rapidly changing uncertain systems, such as CPU utilization on a virtual machine (VM), which frequently changes depending on the tasks running on it, or the bandwidth fluctuation that occurs when the number of users increases. The reasons are as follows. *First*, fuzzy logic can handle uncertainty in predictable environments, because it is based on well-understood principles and the use of imprecise information provided in the form of human language. In contrast, under rapid changes in the various workloads, the existing decision-making algorithms based on multi-constraint optimization with a mathematical model need to know details about resource utilization by the server and details on the network’s condition. *Second*, under the offloading decision strategy in edge computing systems, fuzzy logic–based approaches have lower computational complexity than other decision-making algorithms [17]. Compared with other algorithms, fuzzy logic allows for the consideration of multiple parameters in the same framework. Therefore, it can easily handle a multi-criteria decision-making process to decide where offloaded tasks should run. To apply a fuzzy logic–based approach to an MEO system, Sonmez et al. [17] captured the intuition of a real-world administrator to get an automated management system to solve online problems. The script input parameters under consideration were WAN bandwidth, the length of the incoming tasks, VM utilization on the edge server, the delay sensitivity of the related tasks, MAN delay, local edge VM utilization, and remote edge VM utilization. The offloading decisions made consisted of local, neighboring edge, and cloud servers. In our work, we consider the WLAN environment, and extend the offloading decision to adding a mobile device server.

## Flexible computation offloading in a fuzzy-based mobile edge orchestration

### System model

The MEO under consideration consists of a three-tier hierarchical structure: mobile devices, both an edge server and the edge orchestrator, and a global cloud server. It is assumed that a task can be executed locally on a mobile device. Moreover, the edge orchestrator application runs on the edge servers in a distributed manner.

**Fig. 2:**
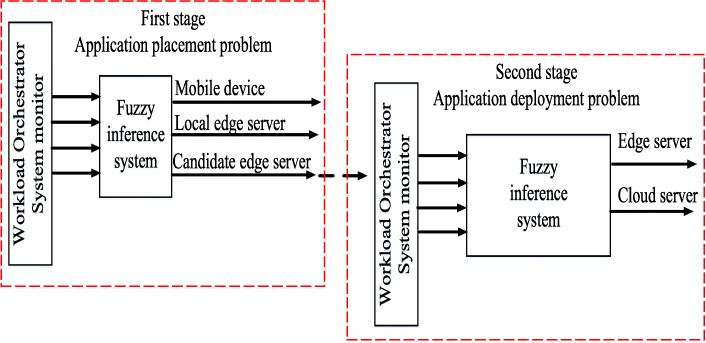
Two-stage decision making marker

The role of the MEO is to determine the target computational unit for an incoming application task by matching application task requirements and network information, such as network bandwidth, and resource availability on edge and cloud servers. By doing so, (1) the MEO will receive the offloaded tasks from the mobile devices, and (2) the target server will be decided upon by using a two-stage decision-making marker [17]. In the first stage, the MEO finds where to place the incoming application task on the edge layer: the mobile device, an edge server, or the nearest edge server. Then, it determines the application deployment that should be executed on the corresponding edge server or cloud server, as shown in Fig. 2.

In this system, we assume that each incoming computation task is independent and can be executed on mobile devices, an edge server, or a cloud server. The decision is made based on a fuzzy logic approach. In order to obtain an efficient decision from the MEO, some requirements and system parameter inputs should be considered. The reasons are discussed as follows. First, the MEC should have enough resource capacity for successful offloading. Second, non-traditional requirements will affect the execution time for the incoming task. Therefore, we consider WLAN delay, MAN delay, local edge VM utilization, the candidate neighboring edge (we call this the candidate edge), and VM utilization for the application placement problem. In the second stage, we consider WAN bandwidth, task length, average VM utilization, and the delay sensitivity of the task. A profile of the application is created to provide the task length, delay sensitivity of the task, and the WLAN delay.

### Packet success ratio versus packet length

**Fig. 3:**
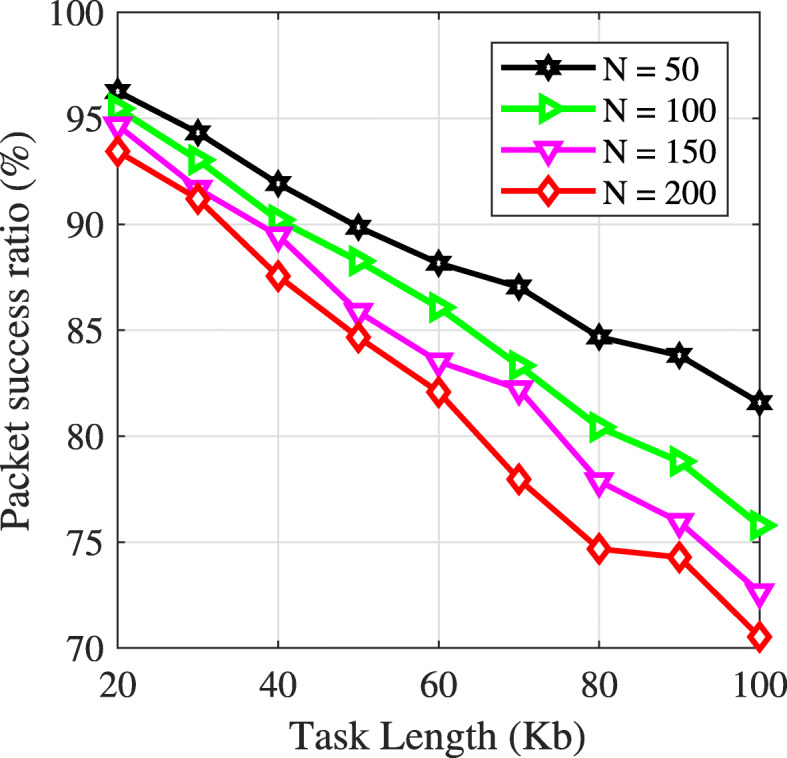
Packet success ratio versus packet length

Following [17], it is assumed that the mobile devices generate tasks during active periods and wait to offload the tasks in idle periods. In this section, we study the packet length’s effect on the packet success ratio under the simulation model in “Performance evaluation” section. From the type of applications (refer to Table 2), the minimum upload/download data sizes is 20Kb. Therefore, we perform the packet length’s effect on the packet success ratio, in which packet length starts with 20Kb. To measure the packet success ratio, one edge server, different packet length, and the number of mobile devices are used. Figure 3 shows the packet success ratio versus packet length. All mobile devices are assumed to have the same packet length. Assuming a fixed transmission rate, when the packet length increases, the packet success ratio decreases. The reason is, the longer the packet length, the longer the transmission time. For instance, the packet success ratios are 75% and 70% for packet lengths of 80 and 100*Kb*, respectively.

**Table 2:** Application types used [17]

	AR	Healthcare	Compute	Info.
Usage percentage (%)	30	20	20	30
Task interval (sec)	2	3	20	7
Delay sensitivity (%)	0.9	0.7	0.1	0.3
Active/idle period (sec)	40/20	45/90	60/120	30/45
Upload/download data (Kb)	1500/25	20/1250	2500/200	25/1000
Task length (GI)	9	3	45	15
VM utilization on Edge (%)	6	2	30	10
VM utilization on Cloud (%)	0.6	0.2	3	1

**Fig. 4:**
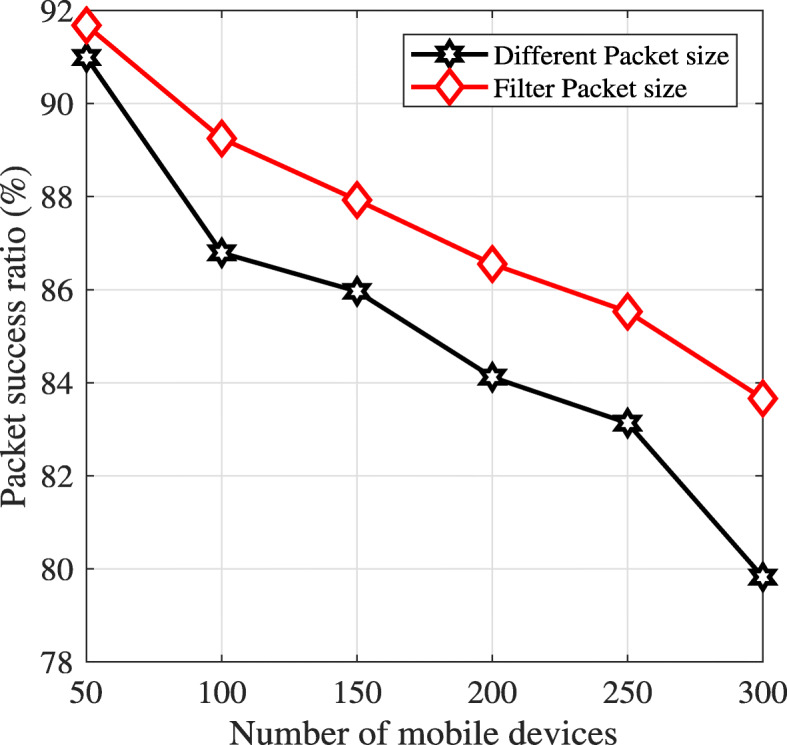
Packet success ratio versus number of mobile devices

Under different packet lengths, the packet success ratio is shown in Fig. 4. When the number of mobile devices increases, the packet success ratio also decreases. The reason is that when the number of mobile devices accesses the channel to transmit application tasks, the collision probability increases [27]. On the other hand, in a MEC system, a task can be executed locally on a mobile device. Therefore, we eliminate the short packet length. It means mobile devices that have a short packet length do not attempt to access the channel to transmit these packets. Compared with a non-filter scheme, the packet success ratio using a filtered packet size is higher, as shown in Fig. 4. As an example, when the number of mobile devices is 300, the packet success ratios using filtered and non-filtered packet lengths are 83.9*%* and 79.7*%*, respectively.

For upper observations, we conclude that under one edge server and different packet lengths, the packet success ratio decreases when the number of mobile devices increases. Therefore, to design an efficient offloading decision, WLAN is an important indicator that has to be considered while offloading tasks to the edge server. For example, if the network congestion is too high, offloading to the edge server is not beneficial.

### Fuzzy logic system for the placement problem

**Fig. 5:**
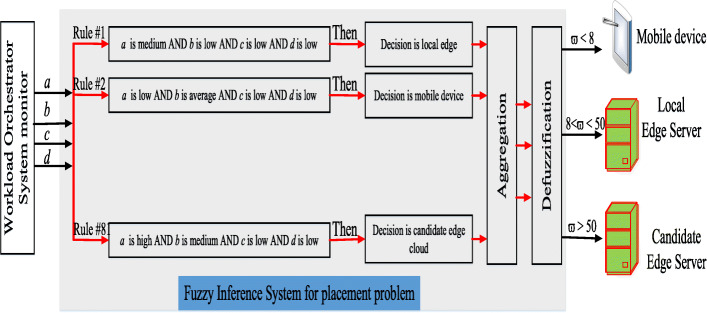
Fuzzy logic system for placement problem

Fuzzy logic can handle uncertainty in predictable environments because it is based on well-understood principles and the use of imprecise information provided in the form of human language. In contrast, under rapid changes in the various workloads, the existing decision-making algorithms based on multi-constraint optimization with a mathematical model need to know details about resource utilization by the server and information on the network’s condition. The components of a fuzzy logic system (FLS) for the placement problem consist mainly of four parts: the fuzzifier, the rules, an inference engine, and the centroid defuzzifier, as shown in Fig. 5. We explain some key mathematical notations in Table 3. Following [15, 17, 25, 26, 28], the operation of the FLS is as follows.

1. Fuzzification is used to transform crisp input sets to fuzzy sets. Note that a crisp set is converted to a linguistic variable (LV) for each indicator. The LV is decomposed into linguistic terms (LTs). We use a membership function (MF) to quantify an LT.

**Table 3:** Definition of key mathematical notations

Symbol	Definition
*Low* (L), *Medium* (M), *High* (H)	Experience level using in the placement problem
*Light* (L), *Normal* (M), *Heavy* (H)	Experience level using in the deployment problem
*μ*^*L*^(*w*)	Membership function, *μ*^*L*^(*w*)={*w*∈*L*,*μ*^*L*^(*w*)∈[0,1]}
*μ*^*M*^(*w*)	Membership function, *μ*^*L*^(*w*)={*w*∈*M*,*μ*^*M*^(*w*)∈[0,1]}
*μ*^*H*^(*w*)	Membership function, *μ*^*L*^(*w*)={*w*∈*H*,*μ*^*H*^(*w*)∈[0,1]}
*a*	WLAN delay
*b*	MAN delay
*c*	Local edge VM utilization
*d*	Candidate edge VM utilization
*x*	WAN bandwidth
*y*	Length of the incoming application task
*z*	VM utilization on the edge server
*t*	Delay sensitivity of the related application
*R*_*i*_	Fuzzy rule at index *i*^*th*^
$\mu ^{R_{i}}_{i}$	The minimum (*min*) function to determine how the results of multiple rules are combined
	within *R*_*i*_. $\mu ^{R_{i}}_{i} = {min}\{\mu _{a}^{R_{i}}(m), \mu _{b}^{R_{i}}(n),\mu _{c}^{R_{i}}(l),\mu _{d}^{R_{i}}(k)\}$
{*m*,*n*,*l*,*k*}	The measured experiment value, as crisp data, is the input parameter to be fuzzified
*μ*_*r*_	The maximum (*max*) function to determine how the results of multiple rules have the same decision *r* on the Fuzzy rules
*ω*	The center of gravity (COG) of the area under the curve by using the centroid defuzzifier method
*T*	Incoming task
*O*	Target of offload
2. Fuzzy input sets are introduced to the inference engine used to evaluate and combine the fuzzy rules from the fuzzy rule base in order to make the inference.
3. The resulting fuzzy output, called a crisp output value, is processed in the defuzzification step by using a centroid defuzzifier method.

*Crisp input variables:* Fuzzy logic systems for the placement problem operate on four crisp input variables, given as

1 $$ F_{1}=\{a,b,c,d\}  $$

where {*a*,*b*,*c*,*d*} are WLAN delay, MAN delay, local edge VM utilization, and candidate edge VM utilization, respectively.

*Linguistic variables:* For {*a*,*b*}, we use *Low* (L), *Medium* (M), *High* (H) as the linguistic variables. For {*c*,*d*}, *Light* (L), *Normal* (M), *Heavy* (H) represent the linguistic variables.

**Fig. 6:**
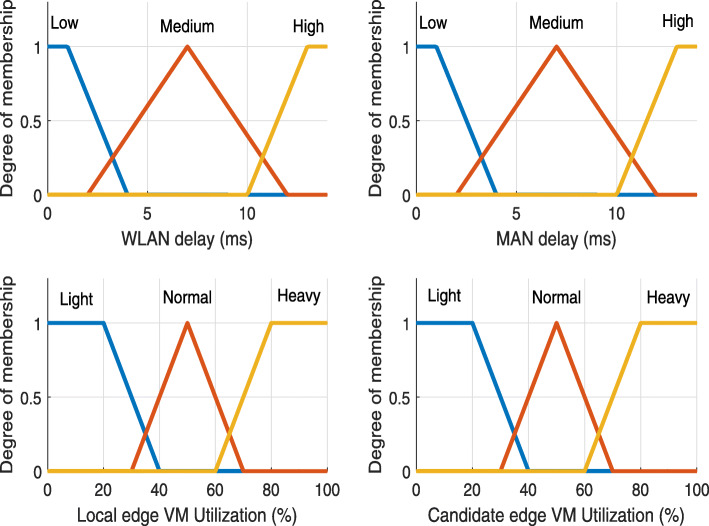
Membership functions used in Fuzzy logic system for placement problem

*Membership function:* In our model, we use the most commonly used one: the triangular form representing the membership function. The values used in membership functions for {*b*,*c*,*d*} were tested in [17]. For the value used in membership function *a*, we conducted various experiments to find the best value. The membership functions of all crisp input variables are depicted in Fig. 6.

We associate a grade with each linguistic term, and the crisp value is transformed into a fuzzy value in the fuzzification step by using these membership functions. They are given as

2 $$  \mathbf{F}_{i}(x) = \left[\mu_{i}^{L}(w),\mu_{i}^{M}(x),\mu_{i}^{H}(x)\right], \text { where} i \in \{a,b,c,d\}  $$

*Fuzzy Rules:* A fuzzy rule is defined as a simple IF-AND-THEN rule with a condition and a conclusion [29]. To determine the fuzzy rules, we vary the relatively better fuzzy rule set that is found empirically, and the best rule combination in the computational experiments is used [17]. The number of fuzzy rules is *n*=3^4^=81 based on four membership functions with three linguistic terms. Table 4 shows example fuzzy rules found empirically for the placement problem

**Table 4:** Example fuzzy rules found empirically for the placement problem

Rule index	*a*	*b*	*c*	*d*	Decision
R1	low	low	light	high	mobile device
R2	low	high	normal	high	mobile device
R3	high	high	normal	low	local edge
R4	medium	medium	heavy	high	local edge
R5	high	high	heavy	high	candidate edge

*Aggregation:* In the aggregation step, we use minimum (*min*) and maximum (*max*) functions to determine how the results of multiple rules are combined within a rule set. We calculate a fuzzy value for selecting the mobile device, the local edge, and the candidate edge server as follows:

3 $$ \mu_{\text{mobile device}}={max} \left\{\mu_{\text{mobile device}}^{R1},..., \mu_{\text{mobile device}}^{Rn}\right\}  $$

4 $$ \mu_{\text{local edge}}={max} \left\{\mu_{\text{local edge}}^{R1},..., \mu_{\text{local edge}}^{Rn}\right\}  $$

5 $$ \mu_{\text{candidate edge}}={max} \left\{\mu_{\text{candidate edge}}^{R3},..., \mu_{\text{candidate edge}}^{Rn}\right\}  $$

where n is 81, and the *min* functions are based on a *Fuzzy Rules* step. For example, in Table 4, the *min* functions are given as

6 $$ \mu_{\text{mobile device}}^{R1}={min}\left\{\mu_{a}^{R1}(m), \mu_{b}^{R1}(n),\mu_{c}^{R1}(l),\mu_{d}^{R1}(k)\right\}  $$

7 $$ \mu_{\text{local edge}}^{R3}={min}\left\{\mu_{a}^{R3}(m), \mu_{b}^{R3}(n),\mu_{c}^{R3}(l),\mu_{d}^{R3}(k)\right\}  $$

8 $$ \mu_{\text{candidate edge}}^{R5}={min}\left\{\mu_{a}^{R5}(m), \mu_{b}^{R5}(n),\mu_{c}^{R5}(l),\mu_{d}^{R5}(k)\right\}  $$

where *m*,*n*,*l*,*k* are the crisp input parameters for the fuzzy inference system.

**Fig. 7:**
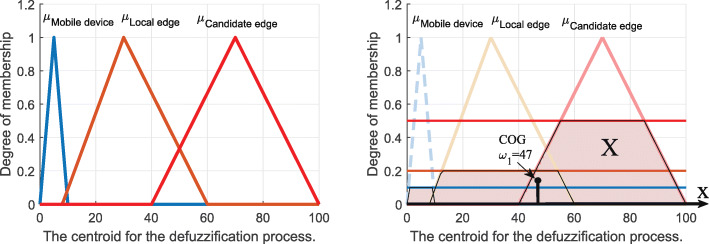
The centroid for the defuzzification process

*Defuzzification:* We use a centroid defuzzifier to calculate the inference. According to [29], the centroid defuzzifier method achieves the center of gravity (COG) of the area under the curve, as shown in Fig. 7. It is calculated as

9 $$ \omega_{1}=\frac{\int_{x \in X} x\mu_{i}(x)}{\int_{x \in X} \mu_{i}(x)},   $$

where *i* is a mobile device, a local edge, or a candidate edge server. After applying the centroid defuzzifier, a crisp output value, *ω*_1_, is in the range [0,100]. Based on *ω*_1_, we define the offloading decision as follows.

10 $$ decision = \left\{\begin{array}{ll} \text{Mobile device}& \text{if}\ \omega_{1} < 8\\ \text{Local edge server} & \text{if}\ 8 < \omega_{1} < 50\\ \text {Candidate edge server} & \text{Otherwise.} \end{array}\right.  $$

### Fuzzy logic system for the deployment problem

Similar to the fuzzy logic system for the placement problem, a fuzzy logic system for the deployment problem operates with the same method, as follows.

*Crisp input variables:* A fuzzy logic system for the deployment problem operates on four crisp input variables, given as

11 $$ F_{2}=\{x,y,z,t\}  $$

where {*x*,*y*,*z*,*t*} are WAN bandwidth, length of the incoming application task, VM utilization on the edge server, and delay sensitivity of the related application, respectively.

*Linguistic variables:* For {*x*,*y*,*t*}, we use *low* (L), *medium* (M), *High* (H) as the linguistic variables. For {*z*}, *light* (L), *normal* (M), *heavy* (H) represent the linguistic variables.

**Fig. 8:**
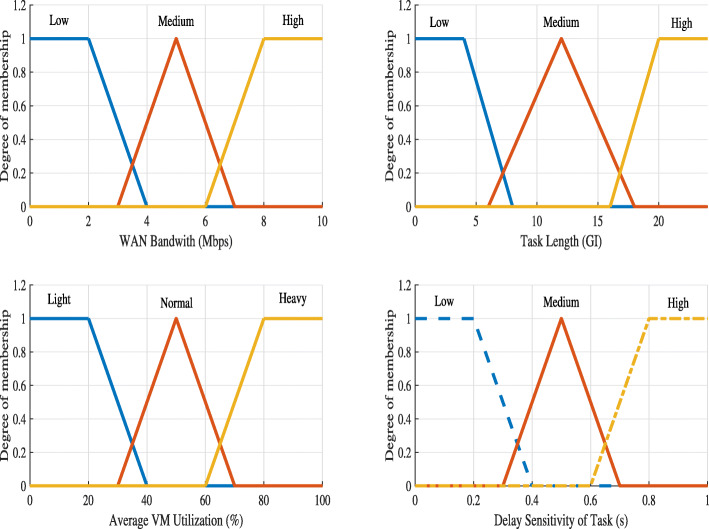
Membership functions used in Fuzzy logic system for deployment problem

*Membership function:* We also use the triangular form to represent a membership function. The membership functions of all crisp input variables are depicted in Fig. 8.

*Fuzzy Rules:* A simple IF-AND-THEN rule with a condition and a conclusion [29] is used in the fuzzy rules. We have the number of fuzzy rules at 3^4^=81 based on four membership functions with three linguistic terms. Table 5 shows example fuzzy rules found empirically for the deployment problem.

**Table 5:** Example fuzzy rules found empirically for the deployment problem

Rule index	*x*	*y*	*z*	*t*	Decision
R1	Low	Low	Light	High	Edge
R2	Low	High	Normal	High	Edge
R3	High	High	Normal	Low	Cloud
R4	Medium	Medium	Heavy	High	Cloud
R5	High	High	Heavy	High	Cloud

*Aggregation:* In the aggregation step, we also use minimum and maximum functions.

**Fig. 9:**
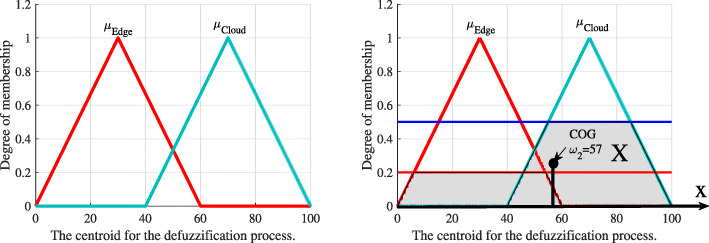
The centroid for the defuzzification process

*Defuzzification:* The centroid defuzzifier method is used to calculate inference, as shown in Fig. 9. It is given as

12 $$ \omega_{2}=\frac{\int_{x \in X} x\mu_{i}(x)}{\int_{x \in X} \mu_{i}(x)},   $$

where *i* belongs to the set for edge processing and cloud processing. Based on *ω*_2_„ we define the offloading decision as follows:

13 $$ decision = \left\{\begin{array}{ll} \text{Edge processing} & \text{if}\ \omega_{2} \leq 50\\ \text {Cloud processing} & \text{Otherwise.} \end{array}\right.  $$

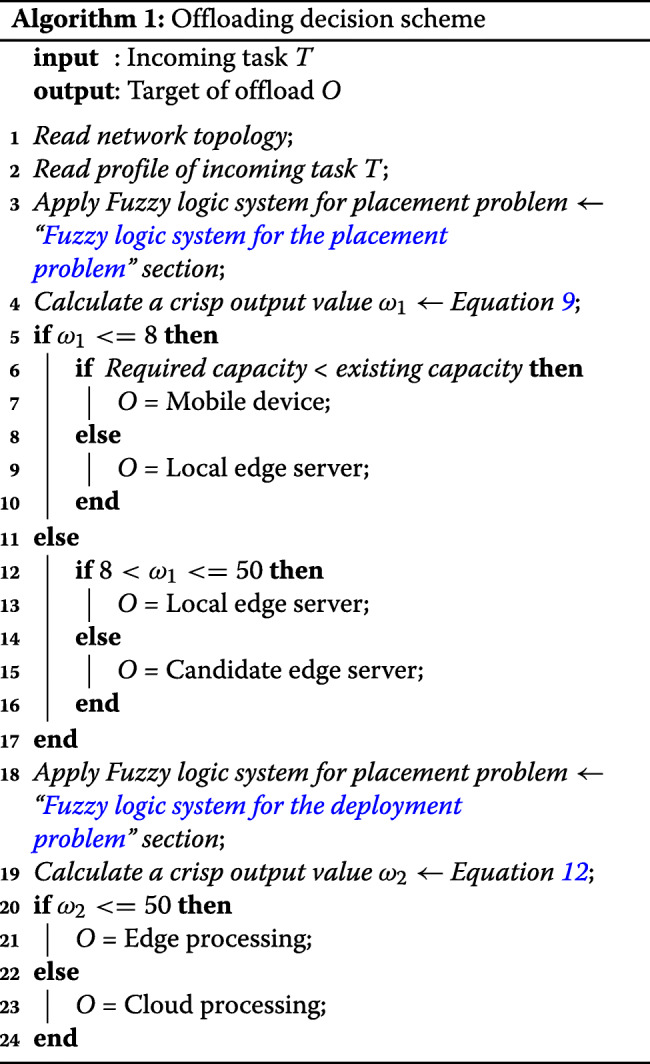


As explained above, the proposed MEO can offload an incoming task to four servers: a mobile device, a local edge, and candidate neighboring edge and cloud servers. We present the details in Algorithm 1.

## Performance evaluation

According to [17, 30–33], the most presented edge computing use cases are considered in our simulation scenario to aim for real-world simulated models. First, an augmented reality application on Google Glass is presented in [31]. Second, the infotainment application is discussed in [32]. Third, a healthcare application that uses a foot-mounted inertial sensor to analyze the walking pattern of the users is studied in [33]. For example, a unmanned aerial vehicle-based smart healthcare system was proposed for Coronavirus disease (COVID-19) monitoring through wearable sensors, movement sensors deployed in the targeted areas [34]. Finally, the example of compute-intensive application is discussed as follows. In the electric bus system, an IoT and cloud network provide updated information for passengers and overall system monitoring [35]. The main objects are to maximize passenger travel during regular bus system implementation and without particular or charter bus requirements, maximize the utilization of buses during its journey, and maximize the utilization of the pre-booked ticket system for better planning [35]. All applications are set up in EdgeCloudSim simulator [30].

In our simulations, we assume that the mobile devices offload tasks which belong to a predefined set of application categories. The user wearing the smart glass offloads captured pictures to the remote servers, which provide face recognition service. The user with the foot-mounted inertial sensor offloads sensor data to the remote servers which provide a fall risk detection service. Similarly, infotainment and the compute-intensive applications send their tasks to the remote servers, which provide related services.

The simulation parameters are presented in Table 6. According to [17], the maximum number of edge servers that can use the network resources is reached because of congestion is 25, which is used in our scenario. The number of mobile users is deployed equally among the edge servers. Each location is covered by a dedicated wireless access point, including edge server, and mobile devices. Moreover, to study all approaches’ performance when the system is overloaded, we vary the number of mobile devices from 200 to 2400. When they move to the related location, they will join WLAN, and they based on their offloading decision send tasks to the edge or cloud server or local processing. Mathematical models compute the WLAN and MAN delays. However, the results are not correct in dynamic environments. To achieve a more realistic simulation environment, the empirical study results for the WLAN and MAN delays are calculated by using values in real-world simulated models. We assume that a single server queue is modeled with Markov-modulated Poisson process (MMPP) arrivals [17]. When the system congestion level is changed, the mean arrival rates of the tasks are updated. Therefore, an empirical study is carried out for characterizing the Internet connection capacity to measure the WLAN/WAN bandwidth. There is not a range of values used in simulations because the WLAN and LAN bandwidths are computed by the average values of the measurements taken at ten consecutive experiments, which are discussed in EdgeCloudSim simulator [17, 30].

**Table 6:** Simulation parameters [17]

*Parameters*	*Value*
Simulation time/warm-up period	33 min / 3 min
Number of edge servers	25
WAN/WLAN bandwidth	empirical
MAN bandwidth	MMPP/M/1 model
LAN propagation delay	5 ms
Number of VMs per edge/cloud server	8/4
Number of cores per edge/cloud VM CPU	2/4 minutes
VM CPU speed per edge/cloud	10/100 GIPS
Mobility model	Random way point
Propagation of selecting a location type	Equal
Number of locations, Type 1/2/3	2/4/8
Mean dwell time in Type 1/2/3	2/5/8 ms

In this paper, we consider the different incoming tasks from four applications: augmented reality, healthcare, compute-intensive, and infotainment applications. They have different profiles in terms of task arrival distribution, delay tolerance, and task size, as shown in Table 2. The inter-arrival time and task size are exponentially distributed random variables [17]. The usage percentage of the application defines how the percentage of mobile devices running this application. We define how frequently the related task is sent to the edge orchestrator by task inter-arrival time, and it follows an exponential distribution. We assume that mobile devices generate tasks during the active period, and they just transmits in the idle period. Data is sent to/received from the server with the upload/download data rate. The delay sensitivity, task length, and VM utilization are used to determine the fuzzy inference system in “Fuzzy logic system for the placement problem” section.

In this paper, we compare four benchmark schemes as follows.

1. *Fuzzy approach:* The fuzzy-based approach [17] considers both computational and communication resources and makes a decision. The MEO finds a target server: either a local edge server, a neighboring edge server, or a cloud server.
2. *Utilization approach:* The CPU utilization–based method will select a target edge server as long as it is not congested in terms of CPU utilization.
3. *Hybrid approach:* The hybrid method considers both WAN bandwidth and the CPU utilization of VMs in the decision process. This method uses threshold values to decide about offloading an incoming task to an edge or cloud server in order to maximize cloud offloading.
4. *Competitor approach*: This approach [15] utilizes fuzzy logic to decide about executing tasks on the mobile device or a cloud server.

**Fig. 10:**
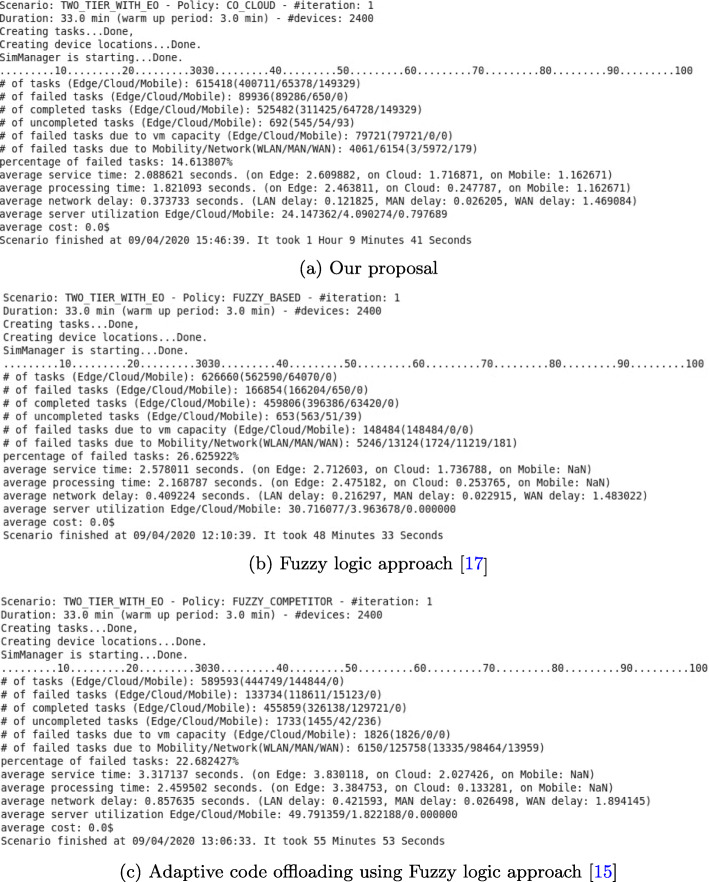
Performance results of three Fuzzy logic approaches with a number of mobile devices is 2400

**Fig. 11:**
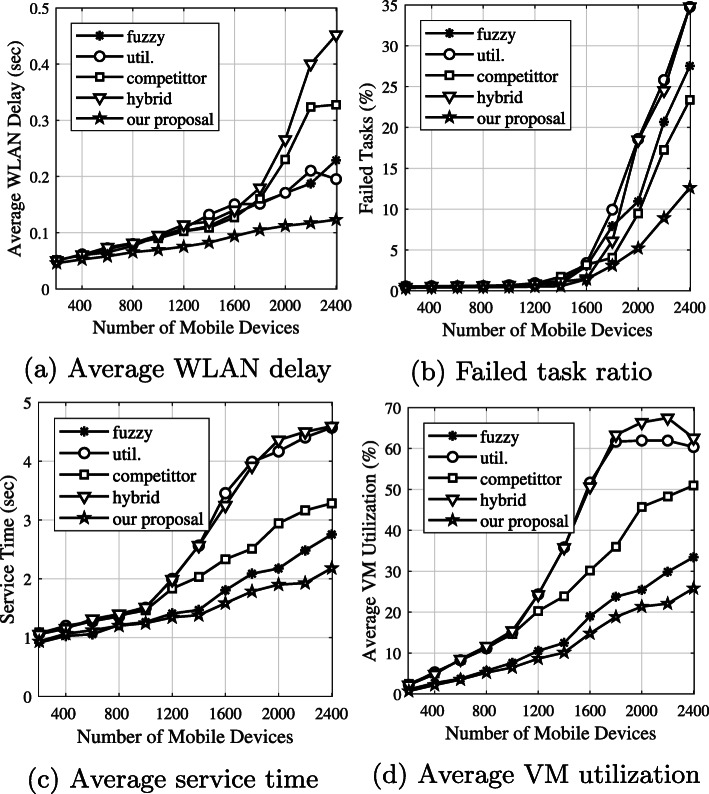
Average WLAN delay, failed-task ratio, service time, and VM utilization based on all application types

We evaluated four performance metrics: average WLAN delay, failed-task ratio, service time, and VM utilization. For instance, performance results described in detail for three fuzzy logic approaches used when a number of mobile devices is 2400 as shown in Fig. 10. Considering the average results based on all application types, Fig. 11 shows the main performance criteria

Figure 11a depicts the average WLAN delay based on all application types. The *hybrid* approach provides the worst performance, because the application tasks will be decided for offload to a cloud server after the CPU resources in the edge server are congested. That means many applications will be in the queue, waiting to be processed on the edge server. This makes the probability of a collision increase in the channel because the application tasks have to be sent again and again until they are successfully received by the edge node. The *fuzzy* approach considers the communication network, MAN delay, and WAN bandwidth. It can balance both computational requirements and the communication network in the task-offloading decision process. It eliminates tasks in the queue for the local edge server by sending them to neighboring edge servers. However, compared with our proposal, we have a trade-off between WLAN delay, and computational and communication edge cloud networks in the decision process. Because a mobile device can execute a short task, the number of application tasks that will be sent to a local edge node decreases. Therefore, our proposal shows better results than its competitors.

Figure 11b shows the average number of unsuccessful application tasks. These tasks consist of i) tasks dropped by the network, and ii) tasks that fail if there are not enough CPU resources on the VM for the incoming task. The *utilization* and *hybrid* approaches decide to offload to the cloud when the computational resources of a VM on edge servers are congested. As a result, some tasks will be dropped by the network due to WAN congestion. Moreover, compared with our proposal, when it comes to other approaches, one of the main reasons for providing poor performance is WLAN congestion. Our proposal provides better results, because we consider the WLAN delay when offloading tasks to an edge server.

The service time consists of network delay and processing delay, as shown in Fig. 11c. As shown in Fig. 11a, our proposal decreases the WLAN delay; therefore, it provides the best performance. The *fuzzy* algorithm balances edge, neighboring edge, and cloud servers. For a low number of mobile devices (less than 1200), our proposal and the *fuzzy* algorithms get approximate results, as shown in Fig. 11a.

Figure 11d shows the average CPU utilization by VMs running on edge servers. This performance result means that if edge servers have lower CPU utilization, the related system is more efficient. As shown in Fig. 11d, our proposal and the *fuzzy* algorithms use the computational resources of VMs better than other algorithms. On the other hand, *utilization* and *hybrid* algorithms decide to offload to a cloud server after the computational resources of the VM are congested. The *competitor* algorithm decides to offload to the edge server when the CPU speed is high. Due to MAN communication failures, these systems could not be utilized well [14]. Our proposal can adapt to dynamic environments more than *fuzzy* algorithms, because it considers not only MAN delay but also WLAN delay. Moreover, a system using our proposal can use the mobile device resources to execute short application tasks. As a result, our proposal utilizes the edge server more efficiently than the four algorithms.

***Average WLAN delay based on each application type***

**Fig. 12:**
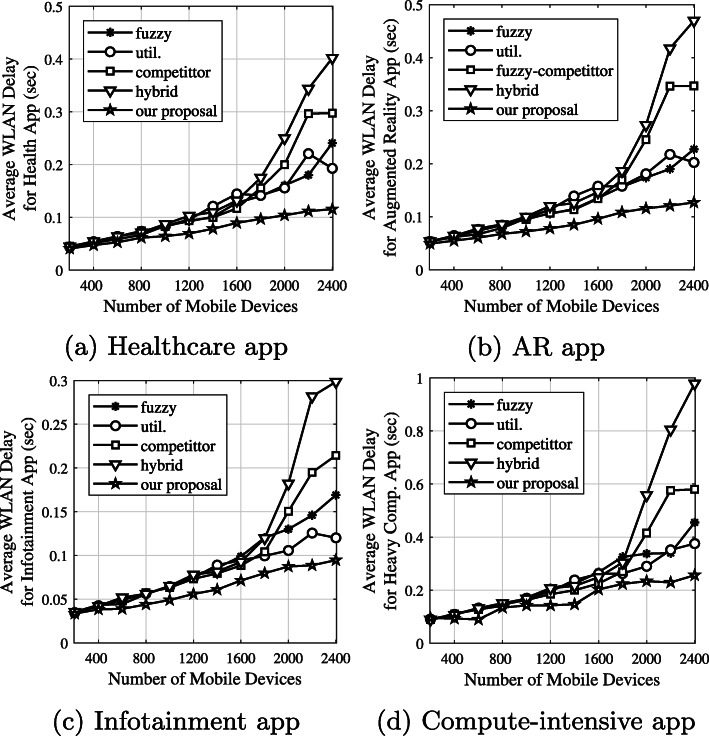
Average WLAN delay based on each application type

We separately analyzed WLAN delays for four applications: VR, augmented reality, healthcare, compute-intensive, and infotainment applications, as shown in Fig. 12. According to Table 2, the task lengths generated are small, medium, big, and very big, for the healthcare, AR, infotainment, and compute-intensive applications, respectively. In our proposal, some short tasks for healthcare applications and medium-length tasks in AR are executed on the mobile device/edge server. Therefore, the number of application tasks sent to an edge node decreased. As a result, WLAN delays in healthcare applications and AR applications gave the best results compared to the competitors, as shown in Fig. 12a and b. Big and very big tasks are sent to edge nodes. However, using our proposal, the system has many chances for more transmissions of application tasks by eliminating short-length tasks. Figure 12c and d show our proposal outperformed its competitors in big and very big tasks.

***Average failed-task rates based on each application type***

**Fig. 13:**
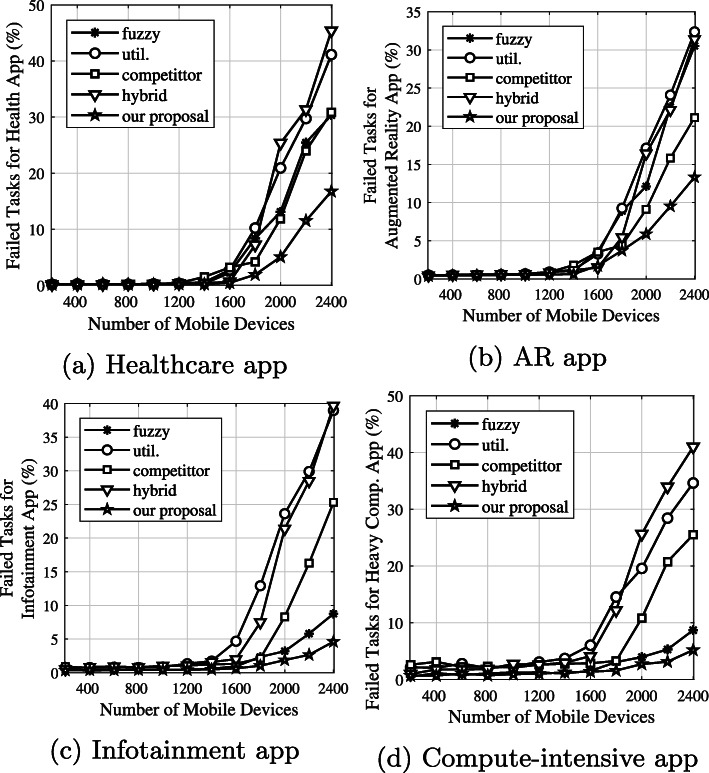
Average number of failed tasks based on each application type

Figure 13 shows the average failed tasks based on each application type. Note that failed tasks are defined as tasks dropped because of network congestion and tasks that fail because of too few CPU resources. When the system is heavy, as when the number of mobile devices is greater than 1600, the performance results between the competitors are different. The failed tasks using the *competitor* algorithm are caused by WLAN and MAN congestion, because that algorithm prefers offloading to edge servers. In systems using the *utilization* and *hybrid* algorithms, the failed tasks are caused by not only WLAN and MAN congestion but also WAN congestion, since they offload to the cloud. Although the *fuzzy* algorithm balances the computational resources and communication network characteristics, it is affected by WLAN congestion. Consequently, by considering WLAN, MAN, and WAN congestion and computational resources, our proposal provides better results based on each application type. On the other hand, small and medium tasks prefer to go to local or neighboring edge servers. Then, the failed tasks happen in the WLAN and MAN environments. Meanwhile, since the big and very big tasks are sent to a cloud server, most of the failed tasks happened in WAN communication. Therefore, the number of failed small and medium tasks (Fig. 13a and b) is higher than for big and very big tasks (Fig. 13c and d).

***Average service time based on each application type***

**Fig. 14:**
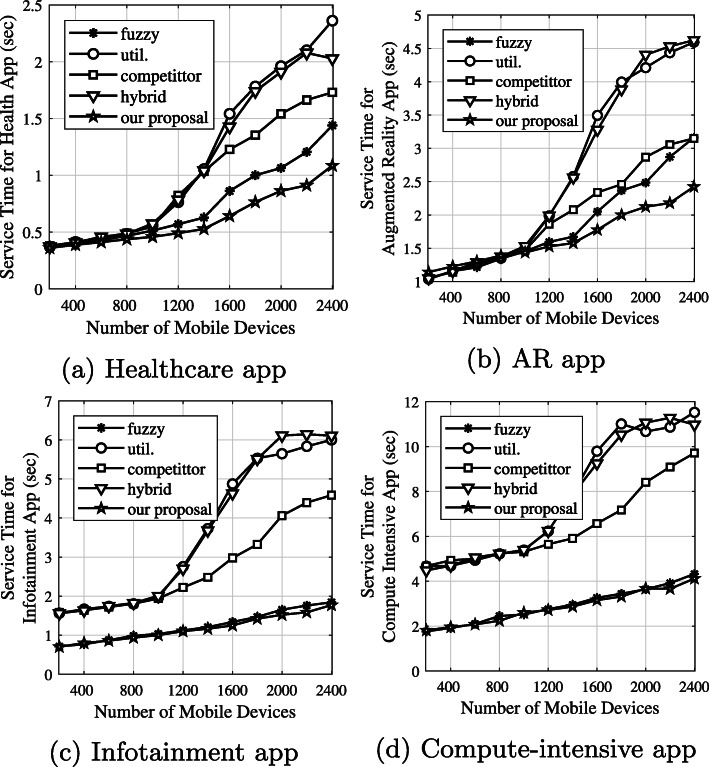
Average service time based on each application type

Figure 14 shows the average service time based on each application type. According to Table 2, the healthcare application has a higher responsiveness level than other applications, and results in 2% CPU utilization on the corresponding edge VM. According to the results in Fig. 14a, our proposal shows better results while serving time-critical small tasks. The reason is that our proposal allows mobile devices to execute a small task without sending it to an edge node. Moreover, WLAN congestion happens when the number of mobile devices is high (e.g., greater than 1000 devices). As a result, the service time of other algorithms is higher than our proposal. In the AR application, our proposal can execute more tasks than its competitors, as shown in Fig. 14b. The reason is that AR applications using our proposal can be executed on mobile edge, local edge, neighboring edge, or cloud servers. Task lengths that are big and very big result in 10% and 30% CPU utilization on the corresponding edge VM, as shown in Table 2. Therefore, these application tasks prefer offloading to cloud servers because the VMs running on the cloud servers are very powerful. Since the *fuzzy* algorithm and our proposal balance the computational resources and communication network characteristics, these performance results are approximate and better than other algorithms, as shown in Fig. 14c and d.

## Conclusions

In this paper, we proposed flexible computation offloading in fuzzy-based mobile edge orchestration for IoT applications, which manages the computing resources to increase performance. Depending on the available information on the network connections and the states of the edge and the cloud, the MEO decides where to offload the incoming client requests to increase the performance. In our system, a fuzzy logic-based workload orchestrator is proposed to provide the efficient offload decision: a mobile device, a local edge, a neighboring edge, or a cloud server, and allocates the edge resources. Our study’s main objective is to solve the bottlenecks of the multi-tier edge computing architectures because of the essential factors: WLAN delay, MAN delay, local and neighboring VM utilizations. These crisp variables are used in fuzzy logic operations to determine the decision for small and medium tasks executed on the mobile user or local edge servers, or a neighboring edge cloud. We set up a simulation environment to evaluate our proposal’s performance by comparing it with four benchmark solutions. According to the simulation results, our proposal provides better results than its competitors for augmented reality, healthcare, compute-intensive, and infotainment applications. In future work, we will apply a genetic algorithm for task scheduling to improve the quality of service.

## Data Availability

Not applicable.
